# An asparagine residue at the N-terminus affects the maturation process of low molecular weight glutenin subunits of wheat endosperm

**DOI:** 10.1186/1471-2229-14-64

**Published:** 2014-03-14

**Authors:** Eleonora Egidi, Francesco Sestili, Michela Janni, Renato D’Ovidio, Domenico Lafiandra, Aldo Ceriotti, William H Vensel, Donald D Kasarda, Stefania Masci

**Affiliations:** 1 DAFNE, Tuscia University, Viterbo, Italy; 2 IBBA, CNR, Milan, Italy; 3 USDA, ARS, WRRC, Albany, CA, USA; 4 Present address: Institute of Plant Genetics (IGV), CNR, Via Amendola 165/A, 70126 Bari, Italy

**Keywords:** Asparaginyl endopeptidase, Gluten protein maturation, Low molecular weight glutenin subunits, Proteomic analysis, Genetic transformation, Transgenic plants, Wheat

## Abstract

**Background:**

Wheat glutenin polymers are made up of two main subunit types, the high- (HMW-GS) and low- (LMW-GS) molecular weight subunits. These latter are represented by heterogeneous proteins. The most common, based on the first amino acid of the mature sequence, are known as LMW-m and LMW-s types. The mature sequences differ as a consequence of three extra amino acids (MET-) at the N-terminus of LMW-m types. The nucleotide sequences of their encoding genes are, however, nearly identical, so that the relationship between gene and protein sequences is difficult to ascertain.

It has been hypothesized that the presence of an asparagine residue in position 23 of the complete coding sequence for the LMW-s type might account for the observed three-residue shortened sequence, as a consequence of cleavage at the asparagine by an asparaginyl endopeptidase.

**Results:**

We performed site-directed mutagenesis of a LMW-s gene to replace asparagine at position 23 with threonine and thus convert it to a candidate LMW-m type gene. Similarly, a candidate LMW-m type gene was mutated at position 23 to replace threonine with asparagine. Next, we produced transgenic durum wheat (cultivar Svevo) lines by introducing the mutated versions of the LMW-m and LMW-s genes, along with the wild type counterpart of the LMW-m gene.

Proteomic comparisons between the transgenic and null segregant plants enabled identification of transgenic proteins by mass spectrometry analyses and Edman N-terminal sequencing.

**Conclusions:**

Our results show that the formation of LMW-s type relies on the presence of an asparagine residue close to the N-terminus generated by signal peptide cleavage, and that LMW-GS can be quantitatively processed most likely by vacuolar asparaginyl endoproteases, suggesting that those accumulated in the vacuole are not sequestered into stable aggregates that would hinder the action of proteolytic enzymes. Rather, whatever is the mechanism of glutenin polymer transport to the vacuole, the proteins remain available for proteolytic processing, and can be converted to the mature form by the removal of a short N-terminal sequence.

## Background

Wheat is the most widely consumed food crop in the world, being processed to give a wide range of products, such as bread, pasta, biscuits, and noodles. This unique versatility is mostly due to gluten proteins, the cohesive mass remaining after washing out the starch granules and water soluble components from a wheat dough. Gluten was first described by Beccari in 1728 (translated by Bailey [1]).

Gluten proteins, belonging to the “prolamin superfamily” [2], are composed of gliadins and glutenins. Gliadins are monomeric proteins, whereas glutenins are polymeric proteins, considered among the largest natural protein molecules [3]. Glutenin polymers are made up of protein subunits of high (HMW-GS) and low (LMW-GS) molecular weight, linked together by intermolecular disulfide bonds. The size and composition of the glutenin polymers play an important role in determining dough rheological properties [4,5].

The HMW-GS are better characterized than LMW-GS, since these latter are much more numerous and heterogeneous. LMW-GS can be classified according to different criteria, based on their primary structure, electrophoretic mobility in SDS-PAGE and N-terminal amino acid sequence. LMW-GS are distinguished into typical types, namely those proteins having a particular primary structure, and gliadin-like types, namely those LMW-GS that are gliadins according to the primary structure, but functionally act like glutenin subunits, due to the presence of an odd number of cysteine residues. The odd number of cysteines enables these gliadin-like proteins to form intermolecular disulfide bonds that incorporate them into glutenin polymer. Based largely on their order of increasing electrophoretic mobility in SDS-PAGE, LMW-GS are classified into B, C and D groups. The B group is composed primarily of typical LMW-GS, whereas the majority of C and D subunits correspond to gliadin-like proteins. The C subunits include mostly α- and γ-gliadin-like LMW-GS, whereas the D group includes ω-gliadin-like proteins, which may be slower moving than B subunits (rev. in [6]).

**Figure 1:**
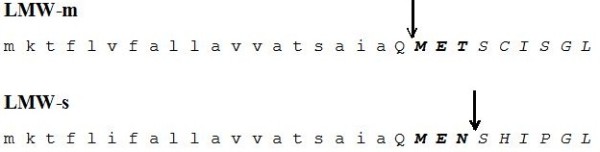
**N-terminal amino acid sequences of the immature LMW-m and LMW-s proteins.** The arrows indicate the start of the mature sequence. The signal peptide is indicated by lowercase letters. Although there is a slight amino acid variation in the two N-terminal sequences of LMW-GS types [11], here only the two sequences considered in the present work are reported.

All these proteins are initially targeted to the endoplasmic reticulum, where signal sequence cleavage occurs, and then transported to the protein storage vacuole. While post-translational modifications of storage proteins have been extensively studied in legumes, they have received less attention in cereals where, with the notable exception of rice, prolamins constitute the majority of the accumulated polypeptides. Identification of distinct processing events would be very useful, not only to understand the mechanisms that lead to accumulation of the mature proteins, but also as a tool to monitor their intracellular transport. Information on post-translational events involved in the biosynthesis of prolamins is indeed rather sparse. For instance, the C-terminal domain of wheat LMW-GS and γ-gliadins presents homology with 2S storage proteins, but, while these proteins are often proteolytically processed after transport to the storage vacuole [7], the wheat proteins maintain an intact C-terminal domain. Proteolytic processing of vacuolar storage proteins is often due to the action of asparaginyl endopeptidases, a class of proteolytic enzymes (also termed vacuolar processing enzymes, VPEs, or legumains) that preferentially cleave after asparagine residues. The repetitive domain of prolamins is rich in proline and glutamine, but poor in asparagine, and thus lacks sites that can be cleaved by these enzymes. However, indication that enzymes belonging to this class may be involved in the maturation of prolamins is now emerging. Cleavage after an asparagine residue has been hypothesized in the processing of certain ω-gliadins [8]. Further evidence indicating that asparaginyl endopeptidases play a role in wheat storage protein maturation is provided by the comparison of the N-terminal sequences of different LMW-GS. Typical LMW-GS are in fact classified according to the N-terminal amino acid residue of the mature protein: serine in LMW-s, methionine in LMW-m and isoleucine in LMW-i types (rev. in [6]). LMW-s and LMW-m are, on the basis of complete nucleotide sequences, practically identical. The N-terminal amino acid sequences of these two protein types differ in that the first three N-terminal amino acids, MET- (or in one minor case, MEN-, [9]) in the mature sequence of LMW-m-types are absent from the amino acid sequence of the s-type. However, the nucleotide sequence encoding these three residues is present in the s-type *lmw-gs* genes with the exception that the codon encoding threonine is replaced by that encoding asparagine, at least in a specific LMW-s type gene (*LMW-42K*) [EMBL:Y17845] [10] (Figure 1).

According to signal sequence cleavage site prediction algorithms, cleavage by signal peptidase would generate a QMET- N-terminal amino acid sequence for LMW-m (or QMEN- in case of LMW-s) type subunits (Figure 1). Removal of the N-terminal glutamine residue would therefore be required to generate m- or s-type LMW-GS, although it has been suggested that the signal cleavage might be degenerate, producing both cleavages [12]. On the other hand, the identification of the gene coding for a specific LMW-s type [EMBL:Y17845] [10] showed the presence of a codon encoding an asparagine in place of a threonine before the start of the mature sequence (MENS- instead of METS-). Accordingly, we postulated that the presence of an asparagine in the unprocessed protein in place of a threonine residue could result in preferential processing to produce an N-terminal end of the LMW-s type [10]. This would result from cleavage of the peptide MEN by an asparaginyl endoprotease, similar to a process that occurs in ω-gliadins [8].

In order to test our hypothesis, we expressed native and mutated versions of LMW-m and LMW-s genes in the endosperm tissue of transgenic durum wheats. Proteomic comparisons, MS/MS analyses and Edman degradation, demonstrated the involvement of asparagine in the formation of the LMW-s glutenin subunits.

Our results indicate that LMW-GS can be quantitatively processed most likely by vacuolar asparaginyl endoproteases, and suggest that LMW-GS accumulated in the vacuole are not sequestered into stable aggregates that would hinder the action of proteolytic enzymes. Rather, our results indicate that, whatever is the mechanism of glutenin polymer transport to the vacuole, the proteins remain available for proteolytic processing, and can be converted to the mature form by the removal of a short N-terminal sequence.

## Results

### Production of wheat transgenic plants

The descriptions of the genes used in this study are reported in the *Plant material and genetic transformation* paragraph (Methods section).

Durum wheat transgenic plants were produced to express native and modified versions of LMW-m (*B1133-WT*, *B1133-T23N*) and LMW-s (*42K-N23T*) type genes in wheat endosperm (Figure 1 and Additional file 1). Eleven, twenty-seven and thirty-two independent regenerated plants (T_0_) were obtained for the *B1133-WT*, *B1133-T23N* and *42K-N23T lmw-gs* genes, respectively, for a transformation efficiency of about 2%, that is within the typical efficiency obtained in stable wheat transformation.

### Proteomic analysis

Since the proteomic patterns of the untransformed durum wheat cultivar Svevo and all the null segregant plants obtained from the progenies of the three types of transgenic plants were identical (data not shown), we eventually used only the null segregant plants as a control for the proteomic comparisons, as in the following description.

### Proteomic comparison between proteins from the plants transformed with the 42K-N23T construct and proteins from the corresponding null segregant plants

**Figure 2:**
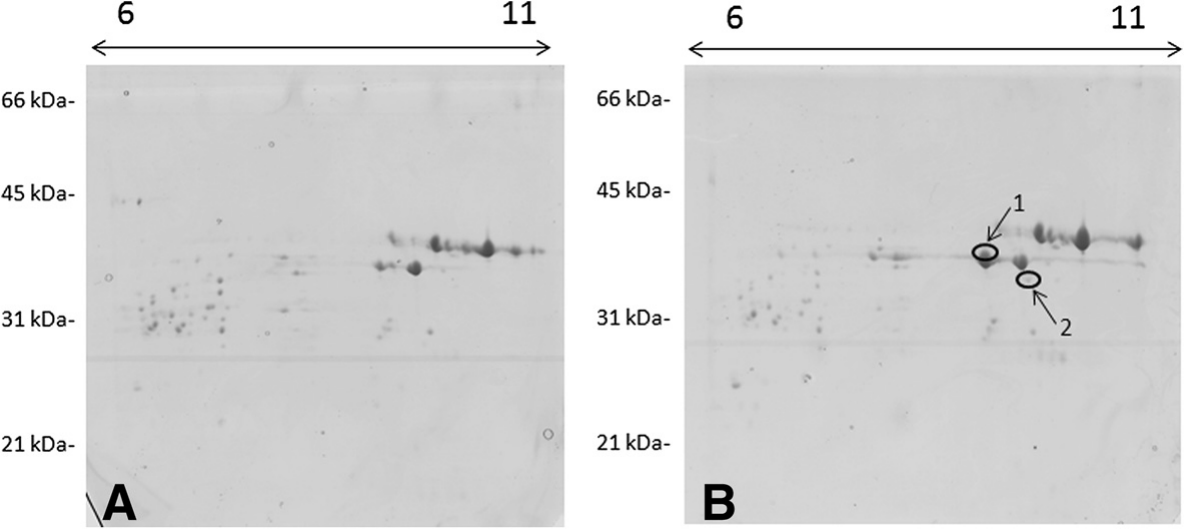
**Comparison between the 2D electrophoretic patterns of B subunits of LMW-GS of the 42K-N23T transgenic lines. A)** null segregant control line; **B)** genotype expressing the *42K-N23T* gene. Arrows point at the two proteins (spots nr. 1 and 2) that are absent in the control line. Molecular weight standards (in kDa) are reported on the left side, and the pI range is indicated at the top of the gels.

The recipient cultivar Svevo possesses a LMW42K protein with pI 8.28 and molecular weight of 42,419, while the expected pI of the 42K-N23T protein is 7.85 with molecular weight of 37,675. Accordingly, we used IPG strips in the pH range 6-11 in order to maximize separation of the native and transgenic proteins. The proteomic comparison of the B subunits obtained from the null segregant control plants and the transgenic plants permitted us to identify two proteins exclusive to the latter (numbers 1 and 2 in Figure 2B).

**Figure 3:**
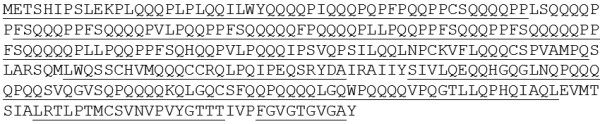
**Complete deduced sequence of the transgenic 42K-N23T protein.** The peptides identified by MS/MS in spot nr. 1 (no discernible sequence was identified in spot. Nr. 2) are underlined.

Analysis of mass spectrometry data from multiple 2D gel separations yielded evidence for the 42K-N23T construct that was identified from 211 total spectra, with 66 exclusive unique spectra, and 54 exclusive unique peptides, resulting in 298 out of 330 amino acids identified, corresponding to 90% sequence coverage, including N-terminal peptides (Figure 3 and Additional file 2). Edman degradation confirmed that the N-terminal amino acid sequence of the transgenic protein present in spot 1 was METSHIP (Table 1). The protein present in spot 2 did not yield sufficient material to provide interpretable N-terminal Edman sequence or tandem mass spectrometry (MS/MS) data, thus the results are available for spot 1 only.

**Table 1:** Comparison between the expected and observed N-terminal amino acid sequences of the eight transgenic proteins

**Genotype**	**Spot n.**	**Expected N-terminal sequence**	**Observed N-terminal sequence**
**42K-N23T**	1	METSHIPSLEKPLQQ-	METSHIP-
**B1133-WT**	3	METSHIFGLERPWQQ-	METSHIFGLERPWQQ-
	4	METSHIFGLERPWQQ-	METSHIFGLERPWQ-
**B1133-T23N**	5	SCIPGLERPWQQQPL-	SCIPGLERP-
	6	SCIPGLERPWQQQPL-	SCIPGLERP-
	7	SCIPGLERPWQQQPL-	SCIPGLERPW-
	8	SCIPGLERPWQQQPL-	SCIPGLERP-

In the “Genotype” column the three transgenic genotypes here analysed are reported. The “Expected N-terminal sequence” column reports the deduced N-terminal amino acid sequences on the basis of transgene nucleotide sequences, whereas the “Observed N-terminal sequence” corresponds to those obtained after Edman degradation, relatively to each of the eight protein spots indicated in the second column.

### Proteomic comparison between proteins from plants transformed with the B1133-WT construct and proteins from the corresponding null segregant plants

**Figure 4:**
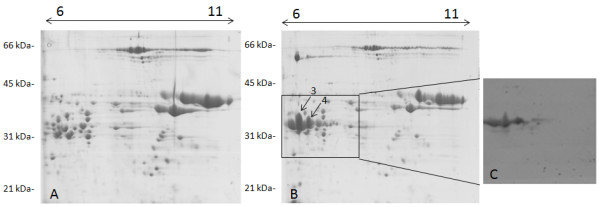
**Comparison between the 2D electrophoretic patterns of glutenin subunits of the B1133-WT transgenic lines. A)** null segregant control line; **B)** genotype expressing the *B1133-WT* gene; **C)** Immunoblotting performed by using the anti-FLAG antibody on the gel region is indicated by the box in **B)**. Arrows point at the two proteins (spots nr. 3 and 4) that are absent in the control line. Molecular weight standards (in kDa) are reported on the left side, and the pI range is indicated at the top of the gels.

**Figure 5:**
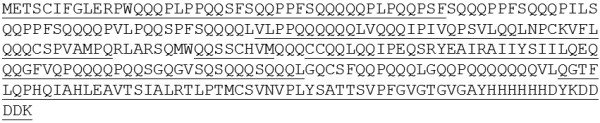
**Complete deduced sequence of the transgenic B1133-WT protein.** The peptides identified by MS/MS in spots nr. 3 and 4 are underlined.

The expected molecular weight of the B1133-WT protein is 34,050 (including tags) and the expected pI is 6.52. Thus, we used IPG strips in the pH range 6-11 on a preparation of total glutenin subunits as a control since this protein is not normally present in the cultivar Svevo. The comparison of gels from transformed and null segregant plants enabled us to identify two main spots present in the transgenic plants, but absent in the control samples. The proteins showed the expected molecular weight, but were visible as what appears to be a charge train (Figure 4B). Immunoblotting performed by using an anti-FLAG antibody to detect proteins of the corresponding gel region gave positive signals matching the same charge train, indicating that the proteins in the spots corresponded to the transgenic proteins (Figure 4C), possibly differing from one another as consequence of glutamine deamidation, which is a common artifact in proteomic analyses [13]. Gel spots were collected and submitted for MS analyses. These results are summarized in Figure 5 and Additional files 3 and 4. The expected sequences are reported and the identified peptides are highlighted or underlined. With Scaffold set to display proteins with a peptide probability of 90%, there were, for example for Spot 3, 38 exclusive unique peptides, 47 exclusive unique spectra, and a total of 239 out of 298 amino acids matched for a coverage of 80%. Both the His- and FLAG tags, as well as the N-terminal peptide, METSCIF-, were identified, confirming that the protein was processed as a LMW-m type (Figure 5 and Additional file 3). Similar results were obtained for spot 4 (Additional file 4). The presence of a phenylalanine residue in seventh position in Spots 3 and 4 instead of the original serine is due to a point mutation that occurred during the cloning procedure.

### Proteomic comparison between proteins of plants transformed with the B1133-T23N construct and proteins from the corresponding null segregant plants

**Figure 6:**
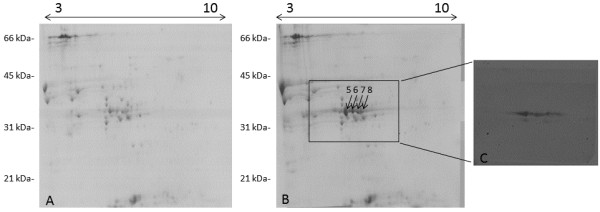
**Comparison between the 2D electrophoretic patterns of glutenin subunits of the B1133-T23N transgenic lines. A)** null segregant control line; **B)** genotype expressing the *B1133-T23N* gene; **C)** Immunoblotting performed by using the anti-FLAG antibody on the gel region indicated by the box in **B)**. Arrows point at the four proteins (spots nr. 5-8) that are absent in the control line. Molecular weight standards (in kDa) are reported on the left side, and the pI range is indicated at the top of the gels.

**Figure 7:**
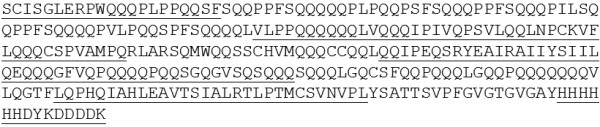
**Complete deduced sequence of the transgenic B1133-T23N protein.** The peptides identified by MS/MS in spots nr. 5-8 are underlined.

As in the case of the B1133-T23N protein, IPG strips in the pH range 3-10 were used to separate a preparation of the total glutenin subunits, since the expected pI was 6.81. Four spots were identified as a possible charge train at the expected molecular weight (33,629) both in the Coomassie stained gels (Figure 6B) and in the corresponding immunoblots performed with the anti-FLAG antibody (Figure 6C). All identified spots were submitted to MS/MS analysis, and corresponded to the same transgenic protein (Additional file 5). For this reason, these results are summarized in Figure 7, in which the expected sequence is reported and the identified peptides are underlined. In summary, 40 total spectra corresponding to 19 unique peptides identified this protein. In total, 159 amino acids were identified, out of 295 (54% coverage), including both the His- and FLAG tags, as well as the N-terminal peptide, that was, as expected, SCISGLE- (Figure 7). These results confirmed that the protein is processed as a LMW-s type. N-terminal amino acid sequencing on four protein spots corresponding to the transgenic proteins, confirmed that the proteins were of the LMW-s types (Table 1).

### Evidence for an N-terminal glutamine

**Figure 8:**
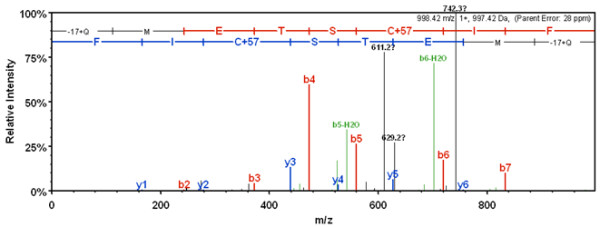
**Fragmentation pattern of the singly charged N-terminal peptide showing evidence for N-terminal glutamine.** Fragmentation pattern of the singly charged N-terminal peptide (mass 998.42), from the B1133-WT construct showing an overlapping series of y- and b- ions for the sequence -ETSCIF (mass 755.32). The first two N-terminal residues of the parent peptide, -17+QM (mass 243.10), were not directly observed in the mass spectrometer, but were deduced from the mass of the parent peptide and the mass of the observed sequence (-ETSCIF). This differential mass was used to elucidate the composition of the N-terminus from tables of known dipeptide masses. The -17 in -17+QM represents a loss in mass as a result of rearrangement of glutamine to pyroglutamic acid.

Examination of a number of MS/MS spectra revealed that, in addition to the peptide METSCIF-, there was another peptide that contained an N-terminal glutamine as evidenced by the presence of a singly charged, protonated peptide of mass 998.42. This peptide fragmented to yield an overlapping series of b and y ions that were interpreted by the sequencing software as the peptide sequence -ETSCIF with an unidentified N-terminal mass of 243.1. This would correspond to the sequence QMETSCIF- in which the N-terminal dipeptide (Q-M) had undergone rearrangement to pyroglutamic acid (Figure 8). The METSCIF peptide was strongly predominant over the QMETSHIF form. A similar N-terminal peptide was identified by DuPont *et al*. [14] in a presumably *Glu-B3* coded LMW-GS from the bread wheat cultivar Butte 86 (both QMET- and QMEN- type sequences were observed) and also by Mamone *et al.*[15] for a LMW-GS, although in this latter case the sequence was QMDT-. The presence of glutamine as first residue corresponds to the expected N-terminal sequence of LMW-GS, according to signal sequence cleavage site prediction. It is of interest that Edman sequencing of the MET-type proteins does not indicate Q as the first amino acid, but rather M, suggesting that the QMETSHIP form cyclizes rapidly and is thus unavailable to Edman sequencing, whereas the amino acid M is readily recognized for MET-type proteins suggesting that cyclization is partial, that the Q is partly removed by some other enzyme (an aminopeptidase?) or the signal cleavage itself is degenerate, producing cleavages before and after the Q [12]. With or without the Q, they both correspond to the LMW-m type. In any case, the reporting of QMET- (found also in this work), QMEN- and QMDT- N terminal peptides indicates that this type of processing may occur in LMW-GS.

Finally, Ikeda *et al.*[9] identified LMW-GS with the N-terminal sequence MENSHIPGLE, likely as the result of a partial escape from the action of asparaginyl endopeptidase. We did not find any MENSHIF in the transgenic polypeptides, although we cannot exclude its presence as a minor component.

## Discussion

Wheat gluten proteins are typical secretory proteins in that their synthesis, folding, maturation and deposition take place within the endomembrane system of the plant cell. Among them, LMW-GS are the most heterogeneous group, being represented by multiple proteins, including those with a characteristic LMW-GS sequence and others with a gliadin-like sequence. In regard to the former, the most common proteins are those typically known as LMW-m and LMW-s types, according to the first amino acid of the mature sequence (methionine or serine, respectively). The LMW-m and LMW-s protein types are practically identical, on the basis of the complete nucleotide sequence, since the main difference resides in the absence or the presence of three additional N-terminal amino acids (MET-, or MEN- in some minor cases [9]) in the mature sequence of LMW-m type, that are instead absent in LMW-s types. However, the corresponding nucleotide triplets are present in the sequences encoding both -s and -m type LMW-GS precursors, except that in one case the third amino acid corresponds to threonine and in the other to asparagine [10]. Because of the presence of either a threonine or an asparagine residue in position 23 of the LMW-m and LMW-s precursors (which corresponds to the third position of the mature LMW-m types), we have hypothesized that, in case of LMW-s types, a preferential (and perhaps secondary) processing at the N-terminal end could occur, that would generate the cleavage of the peptide MEN, most likely by an asparaginyl endoprotease. These proteolytic enzymes have been termed also “legumains” or “vacuolar processing enzymes” [16] and consist of a large family of plant and animal Asn-specific cysteine proteinases. In plants, they occur in storage vacuoles or cell wall of seeds and vegetative organs. In seeds, they play a role in both protein maturation and degradation. They in fact are involved in post-translational processing of protein precursors by cleaving asparagine residues in P1 position of peptide bonds [17]. Asparaginyl endoprotease functions depend on the conformational state of the substrate protein. They are involved in maturation processing when they have access to a normal processing site of the precursor protein, but contribute to the degradation processing by performing an extensive proteolysis, when the protein is misfolded. Thus, seed legumains have a role in protein maturation and also a “structural proof-reading function” [18]. Most of what we know about the characteristics and the biological function of these enzymes is derived from studies on dicot plants. Barley NP1 has been reported to be localized to the cell wall of nucellar cell types [19], while rice REP-2 has been implicated, together with REP-1, in storage protein degradation [20]. These enzymes thus appear to play a role different from storage protein processing. Recently, *OsVPE1*, a rice homolog of the Arabidopsis *βVPE* gene, has been shown to be responsible for the cleavage of rice storage globulins in the protein storage vacuoles [21].

**Figure 9:**
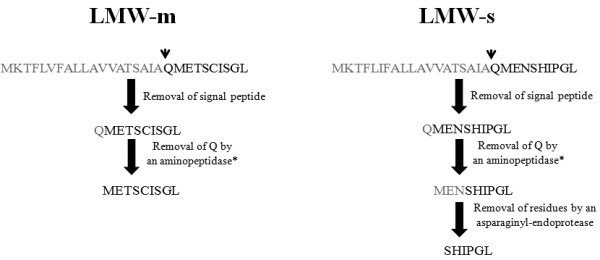
**Proposed model for the differential maturation process of LMW-m and LMW-s.** Arrow-head indicates signal sequence cleavage site as determined by SignalP (http://www.cbs.dtu.dk/services/SignalP/). Asterisk indicates that additional hypotheses on the lack/presence of Q can be proposed (as reported in the sub-paragraph “*Evidence for an N-terminal glutamine*”). Peptides and amino acids removed during the maturation processes are in grey.

In order to determine if the hypothesized maturation processing occurs, we produced durum wheat transgenic plants transformed with three gene constructs, two corresponding to the two *lmw-gs* gene types, namely those with triplets giving rise to threonine or asparagine residues in position 23 of the coding sequence, and a third one in which the codon for the threonine residue was mutated to give rise to an asparagine residue. The transgenic proteins were characterized in T_4_ kernels by means of a proteomic comparison. MS/MS analyses coupled with Edman sequencing of the identified proteins clearly demonstrated that the processing is dependent on the presence of a threonine or asparagine residue in position 23 of the coding sequences: if an asparagine residue is present, proteins are processed as LMW-s types; conversely, when a threonine residue is present, the mature proteins are LMW-m types. In Figure 9 we propose a model for LMW-GS maturation process.

Until now it has not been possible to define whether a low-molecular-weight glutenin subunit gene was a LMW-s or LMW-m type with certainty, because the importance of the asparagine or threonine residue in position 23 of the coding region in the maturation process of LMW-GS had only been hypothesized [10]. However, in different papers, this terminology has already been applied ([22,23] just to cite the most recent ones).

The results here reported are the first direct evidence of the role exerted by asparagine and threonine residues in generating either LMW-m or LMW-s types. It is very likely that a similar maturation process occurs in ω-gliadins, as reported by DuPont *et al.*[8], and also in farinins and triticins, as hypothesized by Kasarda *et al.*[12].

LMW-GS have additional asparagine residues (1 to 3) that are not processed likely because they are protected, either by the presence of proline in P1’ position in some cases, or because they are structurally hidden (sterically inaccessible to the enzyme active site).

While our results do not allow us to infer the compartment in which processing of the N-terminus of LMW-GS occurs, it seems reasonable to assume that such modification occurs after the protein has been transported to protein storage vacuoles. In rice, mutants accumulating storage protein precursors have been very useful in the study of the mechanism of storage protein deposition [24]. Our results indicate that a similar approach may be applied also to wheat.

Finally, although it seems unlikely that the presence/absence of the three amino acids MET- at the N-terminus of LMW-GS can cause differences in grain technological performance, since the main structure of the whole protein is largely unchanged, the plant material described here could be used to perform further analyses in order to assess the role of the changes introduced on dough properties.

## Conclusions

Wheat plant transformation with endogenous genes and site-directed mutated genes, coupled with a proteomic comparison, allowed the determination of the N-terminal maturation process of low-molecular-weight glutenin subunits, and suggest that, in general, LMW-GS can be quantitatively processed most likely by vacuolar asparaginyl endoproteases. This would imply that LMW-GS accumulated in the vacuole do not form stable aggregates, but they remain available for proteolytic processing, necessary for finalizing the maturation process.

## Methods

### Plant material and genetic transformation

The LMW-m and LMW-s type genes and their mutated versions used for wheat transformation were those reported by Ferrante *et al.*[25]. One gene, named *B1133-WT,* corresponds to a native gene that was presumed to code for a LMW-m protein, although it is reported in GenBank as a γ-gliadin [GenBank:M11077] [26]; another one, named *B1133-T23N,* corresponds to the same gene mutated in position 23 to replace a threonine with an arginine. The third gene, named *42K-N23T,* derives from a LMW-s type gene [EMBL:HG529977X], mutated in position 23 to replace an arginine with a threonine. This gene was isolated from genomic DNA of the bread wheat cultivar Yecora Rojo with primers reported in [10]. These three genes were cloned separately into the *Sal*I-*Xba*I restriction sites of pLRPT vector under control of the endosperm-specific HMW-GS Dx5 promoter [27]. The cloning of each gene was achieved by PCR, amplifying the *B1133-WT* or *B1133-T23N* with primers SalHB1133F 5′-acagtcgacatgaagaccttcctcgtcttt-3′ and XbaHisFlagR 5′-tctagatcacttgtcatcgtcatccttgtagtcgtgatggtgatggtgatggt-3′, containing the sequences for the His- and FLAG-tags (see also below), whereas the *42K-N23T* gene was amplified with the primers LMW42KSalF 5′-acagtcgacatgaagaccttcctcatcttt-3′ and LMW42KXbaIR 5′-tctagatcagtaggca ccaactccggt-3′. Since in the past we experienced multiple problems in *LMW-42K* gene [EMBL:Y17845] isolation, mostly due to rearrangements occurring during the cloning procedure because of the particular organization of the repeated domain [10], we deliberately decided not to add tags to this gene construct, which, although helping in protein identification and purification, might contribute to cloning difficulties and/or rearrangements.

PCR reactions were prepared in 50 μl containing 5 μl of 10X FastStart High Fidelity Reaction Buffer (Roche Diagnostics, Monza, Italy), 100 ng of genomic DNA, dNTP Mix 10 mM, 200 ng of each primer, 2.5 units of Fast Start High Fidelity DNA polymerase (Roche Diagnostics, Monza, Italy). The PCR program was: 95°C 2 min, 1 cycle, 95°C 1 min, 62°C 2 min, 72°C 2 min, 30 cycles; 72°C, 5 min. The amplification products were recovered from a 1.2% agarose gel, digested with *Sal*I-*Xba*I and ligated into pLRPT. Constructs were verified by nucleotide sequencing and the *B1133-WT* showed a single substitution which caused the replacement of a serine with a phenylalanine in seventh position of the deduced mature sequence. Despite this substitution, this gene was used because we reasoned it would not interfere with our hypothesis.

The plasmid pAHC20 [28] carrying the *bar* gene that confers resistance to the bialaphos herbicide, was co-bombarded in immature embryos of the durum wheat cultivar Svevo with each of the above LMW-GS genes in a 1:3 molar ratio by following the procedure reported by Volpi *et al.*[29].

Construct schemes are reported in Additional file 1.

### Genomic DNA extraction and PCR analysis of transformed wheat plants

Genomic DNA was extracted from 0.2 g of green tissue as reported in [30].

Transformed T_0_ plants were identified by PCR by using primers specific for the HMW-GS DX5 promoter and the terminator (PRDX5F 5′-catgcaggctaccttccac, PRDX5R 5′-cggtggactatcagtgaattg [31]. The PCR conditions were those reported in the previous paragraph, except for a different extension time that was 1 min and 30 seconds. Positive plants were multiplied up to T_4_ generation, in order to obtain as many homozygous plants as possible to submit to proteomic analyses. Negative plants, corresponding to the null segregant plants, namely those transgenic plants that have lost the transgene by segregation, were also selected, multiplied up to the T_4_ generation, and used as control.

### Proteomic analyses

Plants, either wild type and transgenic lines, included the null segregant genotypes, were grown together in a growth chamber. T_4_ generation plants, previously screened by PCR, were used for proteome analyses, by pooling half seeds of PCR positive plants. Since we were interested only in the presence/absence of the transgenic proteins, we did not use formal replicates, but extracted proteins from each of at least four different positive lines obtained from transformation with each of the three transgenes, and compared to as many biological replicates of the null segregant.

### Extraction of glutenin subunits

Seeds from the durum wheat cultivar Svevo as well from its transgenic lines (included null segregants) were crushed and 50 mg of flour were washed three times with 1 mL of 50% (v/v) propanol in order to remove the soluble protein fraction [32]. In case of extraction of total glutenin subunits, the pellet was eventually extracted with a solution (1 mg: 10 μl) of 50% propanol containing 50 mM Tris-HCl pH 8.8, 1 mM EDTA, 10 mM iodoacetamide or 1.4% of 4-vynilpyridine, 1% (w/v) DTT for 1 h at 70°C. After centrifugation at 13,000 rpm for 15 min, four volumes of cold acetone were added to the recovered supernatant and kept overnight at -20°C to precipitate glutenin subunits. After centrifugation at 13,000 rpm for 15 min, the precipitated proteins were collected and dried in a Savant centrifuge.

In the case of the 42K-N23T protein, since tags were not added, in order to facilitate the identification of differences between null segregant and transformed genotypes, we used as a control a protein fraction enriched in B-type low-molecular weight glutenin subunits (similar in structure to the 42K-N23T protein) that was obtained according to [33].

### 2D electrophoretic analysis (IEF vs SDS-PAGE) of LMW-GS

Quantification of proteins prior to isoelectric focusing (IEF) was performed with the 2-D quant Kit Assay (GE Healthcare).

Total glutenin subunits or B subunits of LMW-GS were suspended in 250 μl of a solution composed of 7 M urea, 2 M thiourea, 4% (w/v) CHAPS, 1.2% (v/v) Destreak Reagent, 0.5% (v/v) IPG buffer pH 3-10 and 6–11 for at least 2 hours. IEF was performed with the *IPGphor™ Isoelectric Focusing System* (Amersham Pharmacia Biotech) and was carried out on immobilized pH gradient (IPG) strips (18 cm, 1 mm) pH 3-10 (for plants transformed with *B1133-WT* and *B1133-T23N* genes) and pH 6-11 (for plants transformed with the *42 K-N23T* gene). The strips were hydrated with samples overnight (12.30 h) at room temperature. IEF was performed at 90,000 volt-hours at 20°C. After focusing, the strips were equilibrated for 30 min at room temperature in a solution of 6 M urea, 2% (w/v) SDS, 30% (v/v) glycerol, 50 mM Tris-HCl, pH 6.8, and 1% (w/v) DTT. Strips were then loaded on the top of a 1 mm thick by 18 cm SDS polyacrylamide gel, T 11%, C 1.28%, by using the *Protean Plus Dodeca cell* (Bio-Rad). Electrophoretic separation was carried out at 40 mA/gel, with cooling at 10°C. Gels were stained with Coomassie Blue G250 [34] and destained in water before image acquisition.

The gel analyses were performed using the software SameSpots Progenesis (vers. 4.5.4293.47197, Nonlinear Dynamics, UK). This software includes statistical analyses such as ANOVA (p ≤ 0.05), and determination of False Discovery Rate (FDR, q ≤ 0.05).

### Western blotting for amino acid sequencing

A 9 cm × 7 cm gel piece, corresponding to the region of interest, namely that including proteins in the molecular weight and pI ranges corresponding to the transgenic proteins, was cut out of the unstained 2D gel and electroblotted on Sequi-blot PVDF (polyvinylidene difluoride) membranes (Bio-Rad, Hercules, CA), previously wetted in methanol and rinsed with deionized water for 5 minutes before soaking in electroblot buffer (10 mM CAPS [3-cyclohexylamino-1-propanesulfonic acid], pH 11). Filter paper (Whatman 3 MM) was also soaked in electroblot buffer before use. Gel pieces were soaked in electroblot buffer for 5-10 minutes. Western blottings were performed using a *Mini Trans Blot Cell* module (Bio-Rad). Transfer was performed for 1 hour at 4°C, at a constant voltage of 100 V. The transfer stack was then dismantled and the membrane was rinsed with distilled water for 5 minutes before staining with Coomassie blue (0,025% (w/v) Coomassie R-250, 40% (v/v) methanol) for 5 minutes. The membranes were then de-stained for 5 minutes in 50% (v/v) methanol, briefly rinsed in distilled water and allowed to air dry at room temperature. Spots of interest were excised using a clean razor blade, and amino acid sequencing was performed essentially according to the procedure reported by Tao and Kasarda [35], but using an Applied Biosystems Procise 492 sequencer.

### Immunoblotting

For immunoblotting experiments, the gel pieces containing the region of interest were incubated in transfer buffer (25 mM Tris- HCl, pH 8,0, 192 mM glycine and 0,04% SDS) for 15 minutes. The blotting was performed in the *Mini Trans Blot Cell* apparatus (Bio-Rad) using a PVDF membrane (Bio-Rad) according to the manufacturer’s protocol. After transferring, the membrane was saturated in 100 ml of Blocking solution (10 mM Tris-HCl pH 8, 150 mM NaCl, 01% Tween20 and 5% non-fat dry milk) at room temperature, on an orbital shaker, for 2 h. The membrane was then washed twice in washing buffer (10 mM Tris-HCl, pH 8, 150 mM NaCl and 0.2% Tween20) and incubated overnight with an anti-Flag-tag polyclonal antibody (Sigma-Aldrich). After removal from the incubation buffer, the membrane was washed extensively and incubated with a horseradish peroxidase-conjugated goat anti-rabbit secondary antibody (Merck Millipore) at room temperature, for 1 hour. The antigen-antibody complex was detected using the “Western blotting Luminol reagent” kit (Santa Cruz Biotechnology, Inc.) following the manufacturer’s procedure.

### Mass spectrometry analysis

Coomassie Brilliant Blue stained bands were cut from the polyacrylamide gels and stored in 1.5 mL Eppendorf tubes. Immediately prior to enzymatic digestion, the gel pieces (1-3) were placed into the wells of a 96-well reaction plate that was positioned in an automated xyz robot (DigestPro, Intavis, Langenfeld, Germany) that automatically destained, reduced, alkylated and digested the proteins in the gel plugs with either chymotrypsin, thermolysin or trypsin. Twenty μg of the selected enzyme was used for each 96-well sample plate and digestion was performed at 35°C. At the end of the digestion period, the instrument eluted the samples of enzymatically cleaved peptides into a 96-well receiving plate that was then inserted into the autosampler interfaced with the QSTAR pulsar *i* hybrid quadrupole-TOF mass spectrometer (Applied Biosystems/MDX Sciex, Toronto, Canada) configured with an electrospray ionization (ESI) source (Protana, Odessa Denmark). Mass spectrometric analysis was performed as previously described [36]. When sufficient material was available the samples were reanalyzed using the data obtained from the first mass spec analysis to form an exclusion list to allow previously unidentified spectra to be analyzed.

The resulting data were searched, using Mascot (http://www.matrixscience.com/) and X!Tandem (http://www.thegpm.org/) against a database containing 11,589 wheat protein sequences from NCBI *T. aestivum* plus a list of common laboratory contaminants (http://www.thegpm.org/) as well as expected sequences from the mutant and wild type expressed proteins. The results of the searches were combined, analyzed and visualized using Scaffold version 4.075 (http://www.proteomesoftware.com).

## Competing interests

The authors declare that they have no competing interests.

## Authors’ contributions

EE carried out the molecular and electrophoretic analyses and made the first draft of the paper; RD and FS contributed to constructs preparation and supervised EE in the molecular analyses; MJ carried out the biolistic transformation that was supervised by RD; AC, DL and DDK critically discussed the results; WHV carried out the MS and Edman analyses and drafted the relevant methods and results; SM conceived the work together with AC, coordinated it and wrote the final draft. All authors read, edited and approved the final manuscript.

## Supplementary Material

Additional file 1 **Plasmids used for biolistic transformation of durum wheat cv. Svevo.** pLRPT vector, containing the *Dx5* promoter and *42K-N23T* (A), *B1133-WT* (B) and *B1133-T23N* (C) genes. Click here for file

Additional file 2 **Data relative to protein spot 1 showing identified sequence and a table showing identified peptides and associated ion statistics.** Individual Excel spreadsheets contain the expected sequences of the protein from protein spot 1: the peptides identified by mass spectrometry are highlighted in yellow, modifications are shown in green: C, carbamidomethyl cysteine; M, oxidation; Q, deamidation. Note that not all cysteine residues are colored green although all have been converted to carboxymethyl amino cysteine. Modification of cysteine was defined as both a fixed and a variable modification in the database searching software. The result is that not all cysteine residues were color-coded by the analysis software. Click here for file

Additional file 3 **Data relative to protein spot 3 showing identified sequence and a table showing identified peptides and associated ion statistics.** Individual Excel spreadsheets contain the expected sequences of the protein from protein spot 3: the peptides identified by mass spectrometry are highlighted in yellow, modifications are shown in green: C, carbamidomethyl cysteine; M, oxidation; Q, deamidation. Note that not all cysteine residues are colored green although all have been converted to carboxymethyl amino cysteine. Modification of cysteine was defined as both a fixed and a variable modification in the database searching software. The result is that not all cysteine residues were color-coded by the analysis software. Click here for file

Additional file 4 **Data relative to protein spot 4 showing identified sequence and a table showing identified peptides and associated ion statistics.** Individual Excel spreadsheets contain the expected sequences of the protein from protein spot 4: the peptides identified by mass spectrometry are highlighted in yellow, modifications are shown in green: C, carbamidomethyl cysteine; M, oxidation; Q, deamidation. Note that not all cysteine residues are colored green although all have been converted to carboxymethyl amino cysteine. Modification of cysteine was defined as both a fixed and a variable modification in the database searching software. The result is that not all cysteine residues were color-coded by the analysis software. Click here for file

Additional file 5 **Data relative to protein spot 5 showing identified sequence and a table showing identified peptides and associated ion statistics.** Individual Excel spreadsheets contain the expected sequences of the protein from protein spot 5: the peptides identified by mass spectrometry are highlighted in yellow, modifications are shown in green: C, carbamidomethyl cysteine; M, oxidation; Q, deamidation. Note that not all cysteine residues are colored green although all have been converted to carboxymethyl amino cysteine. Modification of cysteine was defined as both a fixed and a variable modification in the database searching software. The result is that not all cysteine residues were color-coded by the analysis software. Click here for file
