# Supplementary material for: An asparagine residue at the N-terminus affects the maturation process of low molecular weight glutenin subunits of wheat endosperm
Source: BMC Plant Biol. 2014 Mar 14;14:64. doi: 10.1186/1471-2229-14-64 (PMC4004387; doi:10.1186/1471-2229-14-64)
Supplement: Additional file 1 — Plasmids used for biolistic transformation of durum wheat cv. Svevo. pLRPT vector, containing the Dx5 promoter and 42K-N23T (A), B1133-WT (B) and B1133-T23N (C) genes. [file 1471-2229-14-64-S1.pdf]

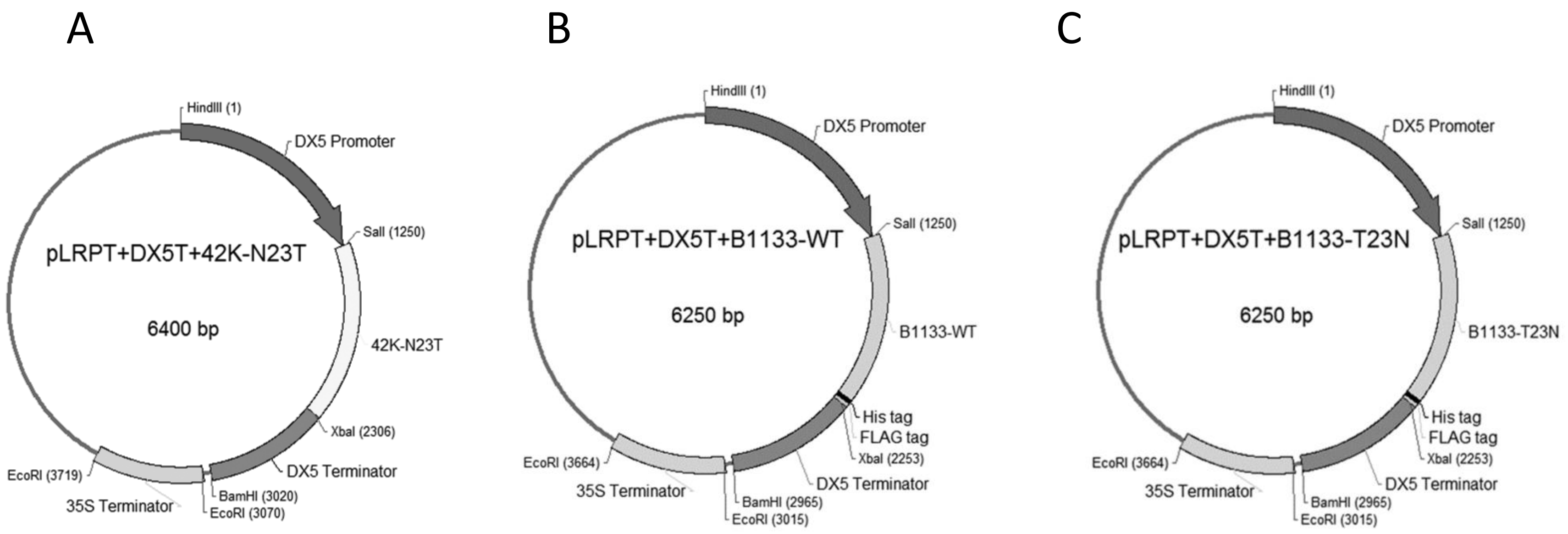

Figure S1. Plasmids used for biolistic transformation of durum wheat. pLRPT vector, containing the *Dx5* promoter and *42K-N23T* (A), *B1133-WT* (B) and *B1133-T23N* (C) genes.
