# Supplementary material for: An asparagine residue at the N-terminus affects the maturation process of low molecular weight glutenin subunits of wheat endosperm
Source: BMC Plant Biol. 2014 Mar 14;14:64. doi: 10.1186/1471-2229-14-64 (PMC4004387; doi:10.1186/1471-2229-14-64)
Supplement: Additional file 2 — Data relative to protein spot 1 showing identified sequence and a table showing identified peptides and associated ion statistics. Individual Excel spreadsheets contain the expected sequences of the protein from protein spot 1: the peptides identified by mass spectrometry are highlighted in yellow, modifications are shown in green: C, carbamidomethyl cysteine; M, oxidation; Q, deamidation. Note that not all cysteine residues are colored green although all have been converted to carboxymethyl amino cysteine. Modification of cysteine was defined as both a fixed and a variable modification in the database searching software. The result is that not all cysteine residues were color-coded by the analysis software. [file 1471-2229-14-64-S2.pdf]

Table S1

Data for spot 1 showing identified sequence (highlighted in yellow) and a table showing identified peptides and associated ion statistics.

Modifications are shown in green: C, carbamidomethyl cysteine; M, oxidation; Q, deamidation. Note that not all cycteine residues are colored green although all have been converted to carboxymethyl amino cysteine. Modification of cysteine was defined in different Mascot searches as both a fixed and a variable modification in the database searching software. The result is that not all cysteine residues were color coded by the analysis software.

42K-VER-130426-mutated-at-23-no-leader (100%), 37,674.7 Da
42K-VER-130426-mutated-at-23-no-leader
54 exclusive unique peptides, 66 exclusive unique spectra, 211 total spectra, 298/330 amino acids (90% coverage)

M E T S H I P S L E   K P L Q Q Q P L P L   Q Q I L W Y Q Q Q Q   P I Q Q Q P Q P F P   Q Q P P C S Q Q Q Q
P P L S Q Q Q Q P P   F S Q Q Q P P F S Q   Q Q Q P V L P Q Q P   P F S Q Q Q Q Q F P   Q Q Q Q P L L P Q Q
P P F S Q Q Q P P F   S Q Q Q Q Q P P F S   Q Q Q Q Q P L L P Q   Q P P F S Q H Q Q P   V L P Q Q Q I P S V
Q P S I L Q Q L N P   C K V F L Q Q Q C S   P V A M P Q S L A R   S Q M L W Q S S C H   V M Q Q Q C C R Q L
P Q I P E Q S R Y D   A I R A I I Y S I V   L Q E Q Q H G Q G L   N Q P Q Q Q Q P Q Q   S V Q G V S Q P Q Q
Q Q K Q L G Q C S F   Q Q P Q Q Q Q L G Q   W P Q Q Q Q V P Q G   T L L Q P H Q I A Q   L E V M T S I A L R
T L P T M C S V N V   P V Y G T T T I V P   F G V G T G V G A Y

Peptide	Amino Acid		Peptide	Mascot			X! Tandem	Number of identified				'+1H Peptide pt	Peptide
Sequence	Prior	Next	Probability	Ion Score	Identity Score	Delta ion Score	-log(e) score	+1H spectra	+2H spectra	+3H spectra	+4H spectra	Mass (AMU) t i	Start index
METSHIPSLEKPL	-	Q	1,00	42,7	34,4	29,5	4,03		3			1481,77	1
METSHIPSLEKPLQQQPLPL	-	Q	1,00	44,7	33	42,2			4			2302,21	1
LQQQPLP	P	L	0,99	42,8	38,4	14	0,05					823,47	13
LQQQPLPLQQ	P	I	1,00	43,7	36,3	17,5	3,40	1				1192,67	13
QQQPLPLQQILW	L	Y	1,00	53,5	34,9	39,2	2,34	2				1491,83	14
ILWYQQQQP	Q	I	1,00	50,2	36,1	35,2	0,72	4				1203,62	23
IQQQPQPFPQQPPCSQQQQPP	P	L	1,00	29,9	29,3	29,8	1,89		9			2486,19	32
FSQQQPPFSQQQQP	P	V	1,00	58,4	32,6	27,7	2,41	2				1674,79	61
VLPQQPP	P	F	0,99	41,2	38,7	12,4	1,24	1				778,45	75
FSQQQQQFPQQQQP	P	L	1,00	35,6	33,7	23,2	1,82	4				1746,82	82
LLPQQPP	P	F	0,98	30,3	38,5		0,21	4				792,46	96
LLPQQPPFSQQQPP	P	F	1,00	34,6	33,8	26,7	0,85	4				1604,84	96
FSQQQQQPP	P	F	1,00	43	37,6	22	1,92	6				1087,52	110
FSQQQQQP	P	L	0,99	32,3	38	13,2	0,29	3				990,46	119
LLPQQPPFSQHQQP	P	V	1,00	49,5	33,6		3,72	14	2			1644,85	127
LPQQPPFSQH	L	Q	1,00				2,82	2				1178,60	128
LPQQPPFSQHQQPVLPQQQIPSVQPSIL	L	Q	1,00	23,8	30,9	5,35	6,34		2	2		3159,70	128
PQQPPFSQHQQP	L	V	1,00				5,62	5				1418,68	129
FSQHQQP	P	V	0,97	34,7	37,4	2,92	1,10	1				871,41	134
SQHQQPVLPQQQIPSVQPSIL	F	Q	1,00	58	33	56	7,55		2			2352,27	135
QQPVLPQQQIPSVQPSIL	H	Q	1,00	39,4	33,9	36,7	3,89		2			2000,12	138
VLPQQQIPS	P	V	1,00	26	37,8	13,2	1,60	9				1009,57	141
VLPQQQIPSVQPS	P	I	0,98				2,09	2				1420,78	141
QQIPSVQPSIL	Q	Q	0,90				0,92	1				1209,68	145
QQLNPCKVF	L	L	1,00	45,9	35,4	26,1	3,04	3				1133,58	156
LNPCKVF	Q	L	0,99	43	38,5	19,1	0,51	5				877,46	158
LQQQCSP	F	V	0,95	35,7	38,9		-2,79	1				860,39	165
LQQQCSPVAMPQ	F	S	1,00				2,59	2				1386,65	165
LWQSSCH	M	V	0,99	25,1	38	5,68	0,75	4				917,39	184
QSSCHVM	W	Q	0,99	39,5	35,1	15,2	0,70	1				848,34	186
VMQQQCCRQLPQIPEQSRYDA	H	I	1,00				3,77		2			2635,22	191
MQQQCCRQLPQIPEQ	V	S	1,00				3,48		1			2057,93	192
CCRQLPQIPEQSRY	Q	D	1,00	0			3,10		2			1834,87	196
RQLPQIPEQSRY	C	D	1,00				4,66	1	1			1514,81	198
LPQIPEQSRYDA	Q	I	1,00	36,7	34,6	7,86	6,85	2				1416,71	200
SIVLQEQQHGQGL	Y	N	1,00	67,3	34,9	17,1	5,46	1	1			1436,75	218
IVLQEQQHGQG	S	L	1,00	48,7	35,6	18,8	5,09	19				1236,63	219
LNQPQQQQPQQS	G	V	1,00	46,9	35,3	39,8	6,77	13				1423,69	230
VQGVSQPQQQQKQ	S	L	1,00	55,2	33,5	48,4	7,22	1				1482,77	242
GVSQPQQQQKQL	Q	G	1,00				4,59	2				1368,72	244
VSQPQQQQKQ	G	L	1,00	74,2	36,5	61,7	5,18	12				1198,62	245
QQQKQLGQCSF	Q	Q	0,99				1,85	1				1351,64	250
LGQCSFQQPQQQQ	Q	L	1,00	60,2	33,7	56,5	5,18	1				1576,72	255
FQQPQQQQ	S	L	1,00	30,5	38	0,56	1,35	2				1031,49	260

**QQPQQQQL**	F	G	1,00	44,2	34,9	9,53	2,27	2	997,51	261
**LGQWPQQQQVPQGT**	Q	L	1,00	35,8	33,7		2,72	4	1594,80	268
**LGQWPQQQQVPQGTL**	Q	L	1,00	52	32,8		3,62	7	1707,88	268
**LLQPHQ**	T	I	0,90	31,6	38,9	1,34	2,54	1	735,41	282
**LQPHQIAQL**	L	E	1,00	32,4	34,8		1,01	2	1047,59	283
**LRTLPTMCS**	A	V	1,00	48,6	37,5	37,2		5	1094,53	299
**RTLPTMCSVNVPVY**	L	G	0,96	42,5	34,6	40,3		1	1652,81	300
**VYGTTT**	P	I	0,90	33,8	36,7	8,01	0,47		641,31	312
**FGVGTGVG**	P	A	1,00	32	38,2	17,1	3,92		693,36	321
**GVGTGVGA**	F	-	0,92				1,26		617,33	322
