# Supplementary material for: An asparagine residue at the N-terminus affects the maturation process of low molecular weight glutenin subunits of wheat endosperm
Source: BMC Plant Biol. 2014 Mar 14;14:64. doi: 10.1186/1471-2229-14-64 (PMC4004387; doi:10.1186/1471-2229-14-64)
Supplement: Additional file 3 — Data relative to protein spot 3 showing identified sequence and a table showing identified peptides and associated ion statistics. Individual Excel spreadsheets contain the expected sequences of the protein from protein spot 3: the peptides identified by mass spectrometry are highlighted in yellow, modifications are shown in green: C, carbamidomethyl cysteine; M, oxidation; Q, deamidation. Note that not all cysteine residues are colored green although all have been converted to carboxymethyl amino cysteine. Modification of cysteine was defined as both a fixed and a variable modification in the database searching software. The result is that not all cysteine residues were color-coded by the analysis software. [file 1471-2229-14-64-S3.pdf]

Table S2

Data for spot 3 showing identified sequence (highlighted in yellow) and a table showing identified peptides and associated ion statistics.

Modifications are shown in green: C, carbamidomethyl cysteine; M, oxidation; Q, deamidation. Note that not all cycteine residues are colored green although all have been converted to carboxymethyl amino cysteine. Modification of cysteine was defined in different Mascot searches as both a fixed and a variable modification in the database searching software. The result is that not all cysteine residues were color coded by the analysis software.

HB1133-WILD_TYPE-LRWT (100%), 34,050.4 Da
Leader-Removed with tag
38 exclusive unique peptides, 47 exclusive unique spectra, 160 total spectra, 239/298 amino acids (80% coverage)

M E T S C I F G L E	R P W Q Q Q P L P P	Q Q S F S Q Q P P F	S Q Q Q Q Q P L P Q	Q P S F S Q Q Q P P
F S Q Q Q P I L S Q	Q P P F S Q Q Q Q P	V L P Q Q S P F S Q	Q Q Q L V L P P Q Q	Q Q Q Q L V Q Q Q I
P I V Q P S V L Q Q	L N P C K V F L Q Q	Q C S P V A M P Q R	L A R S Q M W Q Q S	S C H V M Q Q Q C C
Q Q L Q Q I P E Q S	R Y E A I R A I I Y	S I I L Q E Q Q Q G	F V Q P Q Q Q Q P Q	Q S G Q G V S Q S Q
Q Q S Q Q Q L G Q C	S F Q Q P Q Q Q L G	Q Q P Q Q Q Q Q Q Q	V L Q G T F L Q P H	Q I A H L E A V T S
I A L R T L P T M C	S V N V P L Y S A T	T S V P F G V G T G	V G A Y H H H H H H	D Y K D D D D K

Peptide	Amino Acid		Peptide	Mascot			X! Tandem	Number of identified				+1H Peptide	Peptide
**Sequence**	**Prior**	**Next**	**Probability**	**Ion Score**	**Identity Score**	**Delta ion Score**	**-log(e) score**	**+1H spectra**	**+2H spectra**	**+3H spectra**	**+4H spectra**	**Mass (AMU)**	**Start index**
**METSCIF**	-	G	0,90	51,90	34,20	39,30		2				1837,83	1
**GLERPWQQQPLPPQQSF**	F	S	1,00				7,38		1			951,46	8
**QQQPLPPQQSF**	W	S	1,00	56,20	31,70	46,90			18			948,55	14
**SQQQPILSQQPPFSQQQQPVLPQQSPF**	F	S	1,00				3,14			1		2220,20	52
**PPFSQQQQPVLPQQSPF**	Q	S	1,00				2,62		1			2036,04	62
**SQQQQPVLPQQSPF**	F	S	1,00	55,70	32,10	13,90			2			1537,77	65
**VLPPQQQQQ**	L	Q	1,00				3,52		5			780,39	85
**VLPPQQQQQQL**	L	V	1,00				3,89		7	1		1835,76	85
**VLPPQQQQQQLVQQQIPIVQPSVL**	L	Q	1,00	45,30	26,90	4,30				1	2	1056,60	85
**LVQQQIPIVQPSVL**	Q	Q	1,00				3,96		1			1841,03	95
**VQQQIPIVQPSVL**	L	Q	1,00	74,60	32,00	49,10		1	12			1386,65	96
**PIVQPSVL**	I	Q	0,99				4,49	5				1655,84	101
**VLQQLNPCKVF**	S	L	1,00				5,26		1			1561,93	107
**QQLNPCKVF**	L	L	1,00	43,70	33,60	25,30		2	6			903,36	109
**LQQQCSPVAMPQ**	F	R	1,00				2,10		1			815,45	118
**LQQQCSPVAMPQRL**	F	A	1,00	75,60	31,30	49,70			2			852,52	118
**QQSSCHVM**	W	Q	0,93	43,50	34,50				1			1952,99	138
**QQSSCHVMQQQ**	W	C	1,00				2,70		1			1472,76	138
**CCQQLQQIPEQSRY**	Q	E	1,00				5,85		1			2274,22	149
**QQIPEQSRY**	L	E	1,00	58,50	32,90	42,20			7			1148,57	154
**EAIRAIIY**	Y	S	0,99	47,00	33,90	12,70			6			1133,58	163
**EAIRAIIYSIILQEQQQGF**	Y	V	1,00				15,00			1		1297,65	163
**SIILQEQQQGF**	Y	V	1,00	87,90	33,00	76,40		1	16			976,40	171
**VQPQQQQPQ**	F	Q	1,00				1,77		1			1360,57	182
**VQPQQQQPQQSGQ**	F	G	0,99				1,96		1			878,42	182
**VQPQQQQPQQSGQGVSQSQQQSQQQL**	F	G	1,00				6,62			2		1487,77	182
**GQQPQQQQQQQVL**	L	Q	1,00	69,00	31,70	49,90			2			1650,83	220
**QGTFLQPHQIAHL**	L	E	1,00				7,96		1			1290,67	233
**QGTFLQPHQIAHLEAVTSIAL**	L	R	1,00				2,50			1		3090,56	233
**LQPHQIAHL**	F	E	1,00				2,89		8			1611,81	237
**LQPHQIAHLEAVTSIAL**	F	R	1,00				2,46		3	1		1065,57	237
**PHQIAHL**	Q	E	0,90				2,96		2			1306,71	239
**RTLPTMC**	L	S	0,90				4,11	1				2736,54	254
**RTLPTMCSVNVPL**	L	Y	1,00	61,30	32,90	49,00			12			1345,73	254
**RTLPTMCSVNVPLY**	L	S	1,00				2,57		2			1080,54	254
**CSVNVPLY**	M	S	0,99	36,20	33,70	25,90		1				1480,71	260
**GVGTGVGAY**	F	H	0,98	47,20	34,60	5,87		1				2907,39	276
**HHHHHHDYKDDDDK**	Y	-	1,00	73,30	31,60	58,20				6		1448,85	285
