# Supplementary material for: An asparagine residue at the N-terminus affects the maturation process of low molecular weight glutenin subunits of wheat endosperm
Source: BMC Plant Biol. 2014 Mar 14;14:64. doi: 10.1186/1471-2229-14-64 (PMC4004387; doi:10.1186/1471-2229-14-64)
Supplement: Additional file 4 — Data relative to protein spot 4 showing identified sequence and a table showing identified peptides and associated ion statistics. Individual Excel spreadsheets contain the expected sequences of the protein from protein spot 4: the peptides identified by mass spectrometry are highlighted in yellow, modifications are shown in green: C, carbamidomethyl cysteine; M, oxidation; Q, deamidation. Note that not all cysteine residues are colored green although all have been converted to carboxymethyl amino cysteine. Modification of cysteine was defined as both a fixed and a variable modification in the database searching software. The result is that not all cysteine residues were color-coded by the analysis software. [file 1471-2229-14-64-S4.pdf]

Table S3

Data for spot 4 showing identified sequence (highlighted in yellow) and a table showing identified peptides and associated ion statistics.

Modifications are shown in green: C, carbamidomethyl cysteine; M, oxidation; Q, deamidation. Note that not all cycteine residues are colored green although all have been converted to carboxymethyl amino cysteine. Modification of cysteine was defined in different Mascot searches as both a fixed and a variable modification in the database searching software. The result is that not all cysteine residues were color coded by the analysis software.

HB1133-WILD_TYPE-LRWT (100%), 34,050.4 Da
Leader-Removed with tag
31 exclusive unique peptides, 39 exclusive unique spectra, 118 total spectra, 222/298 amino acids (74% coverage)

M E T S C I F G L E	R P W Q Q Q P L P P	Q Q S F S Q Q P P F	S Q Q Q Q Q P L P Q	Q P S F S Q Q Q P P
F S Q Q Q P I L S Q	Q P P F S Q Q Q Q P	V L P Q Q S P F S Q	Q Q Q L V L P P Q Q	Q Q Q Q L V Q Q Q I
P I V Q P S V L Q Q	L N P C K V F L Q Q	Q C S P V A M P Q R	L A R S Q M W Q Q S	S C H V M Q Q Q C C
Q Q L Q Q I P E Q S	R Y E A I R A I I Y	S I I L Q E Q Q Q G	F V Q P Q Q Q Q P Q	Q S G Q G V S Q S Q
Q Q S Q Q Q L G Q C	S F Q Q P Q Q Q L G	Q Q P Q Q Q Q Q Q Q	V L Q G T F L Q P H	Q I A H L E A V T S
I A L R T L P T M C	S V N V P L Y S A T	T S V P F G V G T G	V G A Y H H H H H H	D Y K D D D D K

Peptide	Amino Acid		Peptide	Mascot			X! Tandem	lumber of identified				+1H Peptide	Peptide
Sequence	Prior	Next	Probability	Ion Score	Identity Score	Delta ion Score	-log(e) score	+1H spectra	+2H spectra	+3H spectra	+4H spectra	Mass (AMU) t	Start index
METSCIF	-	G	0,98	46,4	32,4	38		1				903,36	1
GLERPW	F	Q	1,00	40,9	32,1	20	1,42	1	2			757,40	8
GLERPWQQQPLPPQQSF	F	S	1,00				19,20			1		2036,04	8
QQQPLPPQQSF	W	S	1,00	63	30,3	46,4	5,68		16			1297,65	14
IVNGEEAVPGSWPWQVSLQDKTGF	R	H	1,00				7,74			1		2644,30	16
SQQPPFSQQQQQPLPQQPSF	F	S	1,00	57,2	27,8	57,2	5,02			1		2325,13	25
QVSLQDKTGF	W	H	1,00	73,5	32,1	49,5	3,70		4			1122,58	30
DQGSSSEKIQ	F	K	1,00				4,62		1			1078,50	72
DQGSSSEKIQKL	F	K	1,00	56,4	31,3	46,7	6,85		2			1319,68	72
KIAKVF	L	K	1,00	32,7	33,1	3,53	2,96	1	3			705,47	84
VLPPQQQQQ	L	Q	0,98				3,00		1			1065,57	85
VLPPQQQQQL	L	V	1,00	31,7	31,4	15,8	5,57		4			1306,71	85
VLPPQQQQQLVQQQIPIVQPSVL	L	Q	1,00	50,6	25,4	50,6	-1,97			4	1	2736,54	85
VQQQIPIVQPSVL	L	Q	1,00	51,8	31,2	38,4	3,96		11			1448,85	96
TINNDITLL	L	K	1,00	53,3	31,5	15,2	0,70	2	1			1016,56	98
KLSTAASF	L	S	1,00	53,4	31,7	16,6	1,96	3				824,45	107
QQLNPCKVF	L	L	1,00	39,2	33,9	20,9	4,72	2	7			1133,58	109
LQQQCSPVAMPQ	F	R	0,93				2,16		1			1386,65	118
QQSSCHVM	W	Q	1,00	60	33,8		2,77		1			976,40	138
ANTPDRLQQA	N	S	0,98				3,04		1			1113,56	149
ANTPDRLQQASLPL	N	L	0,98				3,35		1			1523,82	149
CCQQLQQIPEQSRY	Q	E	1,00				9,01		1			1837,83	149
QQIPEQSRY	L	E	1,00	59	31,7	47,8	6,48		4			1148,57	154
EAIRAIIY	Y	S	1,00	40,5	32,7	11,5	3,48	2	9			948,55	163
EAIRAIIYSIILQEQQQGF	Y	V	1,00				9,40			1		2220,20	163
SIILQEQQQGF	Y	V	1,00	84,6	32,3	74,8	-2,61		1			1290,67	171
GTKIKDAM	W	I	0,99	25,6	34	12,2	2,11		1			863,47	173
VQPQQQQPQQSGQ	F	G	0,99				4,10		1			1480,71	182
VQPQQQQPQQSGQGVSQSQQQSQQQL	F	G	0,97	19	25	7,97	1,92				1	2907,39	182
GDSGGPL	M	V	1,00	39,2	32,2	7,74	2,40	2				602,28	193
TLVGIVSW	W	G	1,00	42,5	32,7	31,6	2,37	2	1			874,50	208
VGIVSW	L	G	0,95	32,5	32,3	11,1	0,04	1				660,37	210
ARVTALVNWVQQTL	Y	A	0,98				3,41		4			1598,90	229
QGTFLQPHQIAHLEAVTSIAL	L	R	0,97				4,11			2		2257,20	233
LQPHQIAHL	F	E	1,00	40,9	32,4	15,1	2,00		4			1056,60	237
LQPHQIAHLEAVTS	F	I	1,00				8,64		1			1543,82	237
EAVTSIAL	L	R	0,99	50,3	32,5	18,4		2				803,45	246
RTLPTM	L	C	0,93	18,9	33,4		0,89	1				718,39	254
RTLPTMCSVNVPL	L	Y	1,00	61,7	32	48,8	-2,54		7			1487,77	254
RTLPTMCSVNVPLY	L	S	0,99				3,82		4			1650,83	254
SATTSVPF	Y	G	0,99	27,8	32,6		0,77	2				809,40	268
