# Supplementary material for: An asparagine residue at the N-terminus affects the maturation process of low molecular weight glutenin subunits of wheat endosperm
Source: BMC Plant Biol. 2014 Mar 14;14:64. doi: 10.1186/1471-2229-14-64 (PMC4004387; doi:10.1186/1471-2229-14-64)
Supplement: Additional file 5 — Data relative to protein spot 5 showing identified sequence and a table showing identified peptides and associated ion statistics. Individual Excel spreadsheets contain the expected sequences of the protein from protein spot 5: the peptides identified by mass spectrometry are highlighted in yellow, modifications are shown in green: C, carbamidomethyl cysteine; M, oxidation; Q, deamidation. Note that not all cysteine residues are colored green although all have been converted to carboxymethyl amino cysteine. Modification of cysteine was defined as both a fixed and a variable modification in the database searching software. The result is that not all cysteine residues were color-coded by the analysis software. [file 1471-2229-14-64-S5.pdf]

Table S4

Data for spot 5 showing identified sequence (highlighted in yellow) and a table showing identified peptides and associated ion statistics.

Modifications are shown in green: C, carbamidomethyl cysteine; M, oxidation; Q, deamidation. Note that not all cycteine residues are colored green although all have been converted to carboxymethyl amino cysteine. Modification of cysteine was defined in different Mascot searches as both a fixed and a variable modification in the database searching software. The result is that not all cysteine residues were color coded by the analysis software.

HB1133T23N_After (100%), 33,628.8 Da
ASN cleavage
19 exclusive unique peptides, 21 exclusive unique spectra, 40 total spectra, 159/295 amino acids (54% coverage)

```
S C I S G L E R P W   Q Q Q P L P P Q Q S   F S Q Q P P F S Q Q   Q Q Q P L P Q Q P S   F S Q Q Q P P F S Q
Q Q P I L S Q Q P P   F S Q Q Q Q P V L P   Q Q S P F S Q Q Q Q   L V L P P Q Q Q Q Q   Q L V Q Q Q I P I V
Q P S V L Q Q L N P   C K V F L Q Q Q C S   P V A M P Q R L A R   S Q M W Q Q S S C H   V M Q Q Q C C Q Q L
Q Q I P E Q S R Y E   A I R A I I Y S I I   L Q E Q Q Q G F V Q   P Q Q Q Q P Q Q S G   Q G V S Q S Q Q Q S
Q Q Q L G Q C S F Q   Q P Q Q Q L G Q Q P   Q Q Q Q Q Q Q V L Q   G T F L Q P H Q I A   H L E A V T S I A L
R T L P T M C S V N   V P L Y S A T T S V   P F G V G T G V G A   Y H H H H H H D Y K   D D D D K
```

Peptide	Amino Acid		Peptide	Mascot			X! Tandem	Number of identified				'+1H Peptide	Peptide
Sequence	Prior	Next	Probability	Ion Score	Identity Score	Delta ion Score	-log(e) score	+1H spectra	+2H spectra	+3H spectra	+4H spectra	Mass (AMU)	Start index
**SCISGLERPW**	-	Q	0,997	59,2	42	31,6	4,28		2			1204,58	1
**SCISGLERPWQQQPLPPQQSF**	-	S	0,997				9,3			2		2483,21	1
**QQQPLPPQQSF**	W	S	0,996	22,3	22,3	30,3	3,4		1			1297,65	11
**VLPPQQQQQL**	L	V	0,997	31,9	12,6	31,4	4		2			1306,71	82
**VLPPQQQQQLVQQQIPIVQPSVL**	L	Q	0,997	64,4	64,4	28,2	2,38				2	2736,54	82
**VQQQIPIVQPSVL**	L	Q	0,997	52,2	37,5	31,2	4,19		3			1448,85	93
**VQQQIPIVQPSVLQQLNPCKVF**	L	L	0,997				7,37			2		2563,41	93
**QIPIVQPSVLQQLNPCKVF**	Q	L	0,997				5,57			1		2208,22	96
**QQLNPCKVF**	L	L	0,992				6,24		4			1116,55	106
**LQQQCSPVAMPQ**	F	R	0,914				2,85		1			1386,65	115
**QQIPEQSRY**	L	E	0,997	57,9	47,4	31,7			1			1148,57	151
**EAIRAIIY**	Y	S	0,985	49,7	13,2	32,7			3			948,55	160
**EAIRAIIYSIILQEQQQGF**	Y	V	0,997				11,5			2		2220,20	160
**SIILQEQQQGF**	Y	V	0,997	80,6	69,1	32,3	5,6		4			1290,67	168
**VQPQQQQPQQSGQGVSQSQQQ**	F	S	0,983				4,33			1		2323,10	179
**LQPHQIAHLEAVTSIALRTLPTMCSV**	F	N	0,956				3,31				1	2886,53	234
**RTLPTMCSVNVPL**	L	Y	0,997	55	44,2	32	1,09		3			1487,77	251
**HHHHHHDYKDDDD**	Y	-	0,997				9,77			3		1707,66	282
**HHHHHHDYKDDDDK**	Y	-	0,997	56,9	56,9	29,1	6,24			2		1835,76	282
